# Supplementary material for: BMP signaling regulates dorsal skeletal growth in the sea urchin embryo
Source: Development. 2026 Apr 23;153(16):dev205344. doi: 10.1242/dev.205344 (PMC13200730; doi:10.1242/dev.205344)
Supplement: Supplementary information [file develop-153-205344-s1.pdf]

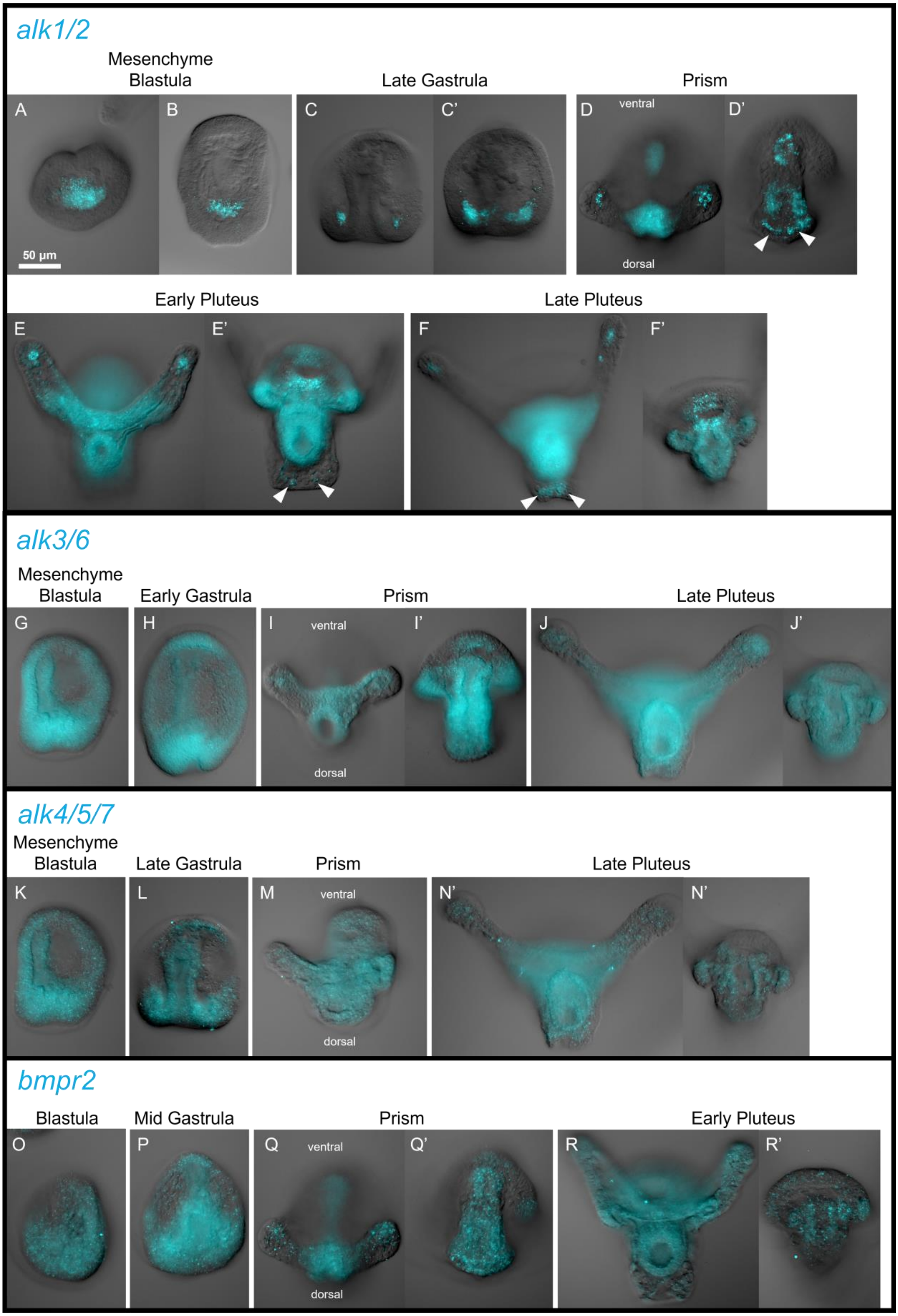

Development • Supplementary information

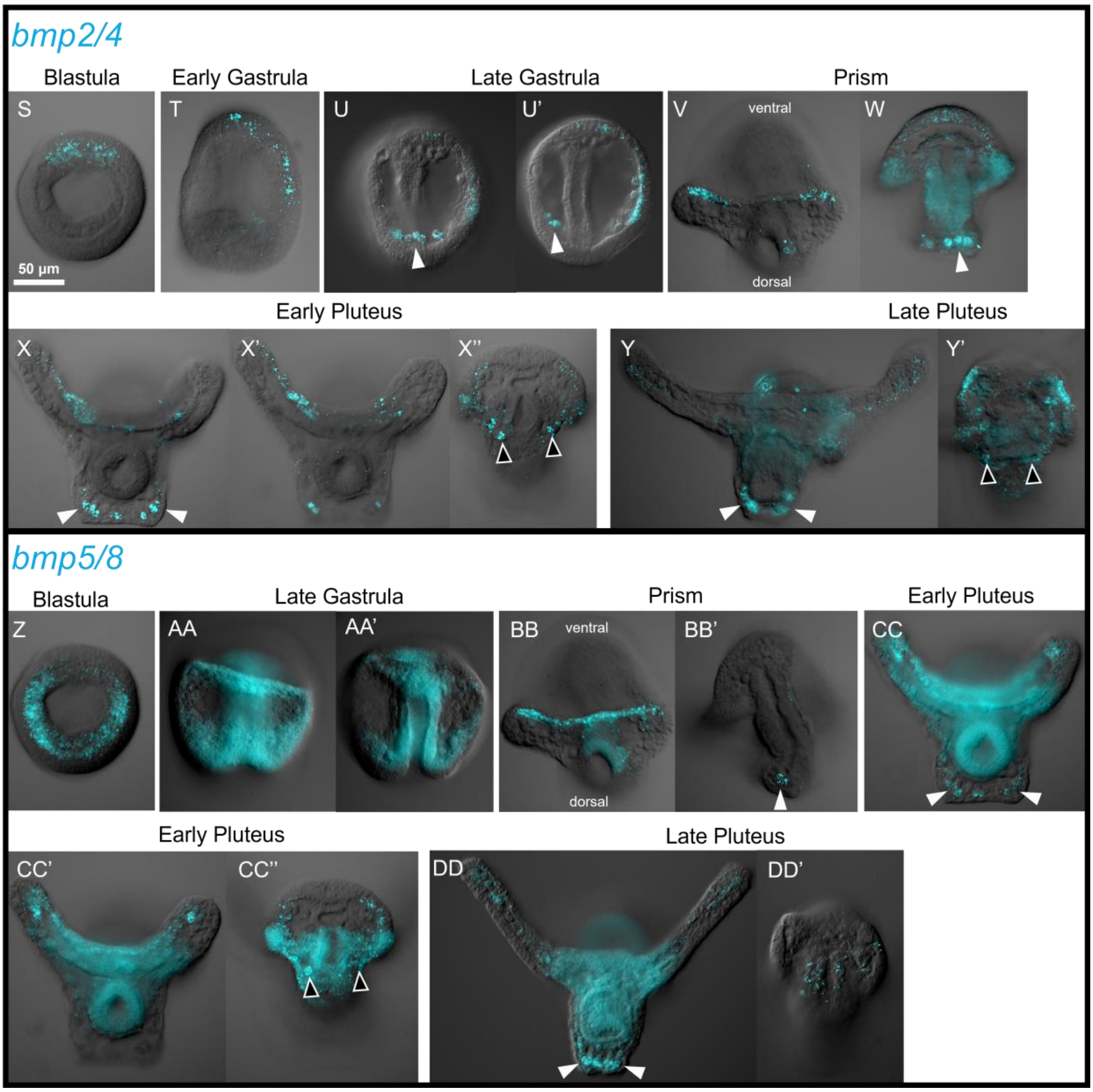

Fig. S1. Spatiotemporal expression pattern of components of the BMP signaling pathway.

Fixed *L. variegatus* embryos from various stages were stained via HCR in-situ hybridization. White arrowheads mark the dorsal region of the skeletal syncytium when expression was visible. Outline arrowheads mark PMCs that are in the recurrent rod region of the skeletal syncytium when expression was visible.

A – C': *alk1/2* was expressed in all of the PMCs at the mesenchyme blastula stage through the gastrula stage. D & D': *alk1/2* expression was then restricted within the PMC syncytium to just the dorsal region and the PMCs at the tips of the post-oral arms at the prism stage. At this same time, *alk1/2* was transiently expressed in

multiple gut domains. E – F’: Through the rest of development, *alk1/2* was expressed in the dorsal region of the syncytium and the PMCs at the tips of the post-oral arms. It was also expressed strongly in two domains on opposite sides of the oral hood along the animal-vegetal axis.

G – J’: Alk3/6 showed extremely weak and ubiquitous expression in all germ layers from mesenchyme blastula stage through the rest of development. There was some enriched expression in the oral hood of the ciliary band at the prism stage (I’).

K – N’: *alk4/5/7* showed moderate ubiquitous expression in all germ layers from the mesenchyme blastula stage to the pluteus stage, which was consistent with previous reports (Piacentino et al., 2015).

O & P: *bmpr2* was moderately expressed in all germ layers ubiquitously from the mesenchyme blastula to the early gastrula stage. Q & Q’: by the prism stage, *bmpr2* expression showed more restriction and was strongly expressed throughout the gut and all three germ layers of the dorsal sheitel of the embryo. At the same time, it was also expressed in the ectodermal region overlying the growing tips of the post-oral arms. R & R’: *bmpr2* expression was still weakly to moderately present in all three germ layers and was still clearly present in the oral hood, coelomic pouchs, and esophagus.

S & T: As previously reported (Duboc et al., 2004), *bmp2/4* was initially expressed throughout the ventral ectoderm up and remained expressed this way during early gastrulation. U & U’: by the end of gastrulation, *bmp2/4* expression began to appear in the dorsal region of the PMC syncytium and its ventral ectodermal expression became more restricted. V & W: *bmp2/4* expression was highly enriched in the dorsal region of the PMC syncytium, the ventral ectoderm overlying the post-oral PMCs, and throughout most of the ciliary band. X – Y’: *bmp2/4* expression remained in the dorsal region of the PMC syncytium throughout the rest of development and appeared in the two PMC bodies at the distal tips of the recurrent rods. It was expressed along the entire ventral ectoderm of the post-oral arms and through most of the oral hood. Interestingly, it was absent from the most animal and vegetal regions of the oral hood where *alk1/2* was typically expressed at these stages.

Z – AA’: finally, *bmp5/8* showed a somewhat ubiquitous expression pattern from the mesenchyme blastula stage through gastrulation, but it more strongly expressed

Development • Supplementary information

where *bmp2/4* was not present (S & Z show the same embryo). BB & BB' the ubiquitous expression of *bmp5/8* was gone by the prism stage and it was only just starting to be expressed in the dorsal region of the PMC syncytium. It was also strongly expressed along the entire ventral-vegetal region of the ciliary band, unlike *bmp2/4* which was restricted to just the ectoderm overly the post-oral arms. CC – CC": at the early pluteus stage, *bmp5/8* became strongly expressed throughout the dorsal region of the PMC syncytium and appeared in the single distal-most PMCs of the recurrent rods. It was also strongly expressed in the distal-most PMC of each post-oral arm. Similar to *bmp2/4*, *bmp5/8* was expressed throughout the oral hood except where *alk1/2* was expressed. DD & DD': at the late pluteus stage, expression of *bmp5/8* weakened, though was not gone, in most domains except the dorsal region of the PMC syncytium where it remained strongly expressed.

The embryos showed strong autofluorescence in the gut at later stages, which was exacerbated by the body being compressed on the slide. As such, this autofluorescence could reach intensity levels similar to punctate HCR staining and could not be eliminated.

At least 30 embryos of each stage for each gene of interest were observed and representative images showing expression patterns consistent with >90% of observed embryos are shown.

Many images shown in Fig. S1 use the same images shown in Fig. 4 of the main manuscript, but provide a view of the entire embryo to show all staining patterns rather than magnified views of particular regions. The following are the images of Fig. 4 and the respective image of Fig. S1 that are the same: Figs 4A & S1A; Figs 4B & S1C'; Figs 4C & S1D; Figs 4D & S1E'; Figs 4E & S1F; Figs 4E' & S1F'; Figs 4F & S1P; Figs 4G & S1Q'; Figs 4J & S1U; Figs 4K & S1W; Figs 4L & S1X; Figs 4L' & S1X'; Figs 4N & S1AA; Figs 4O & S1BB'; Figs 4P & S1CC; Figs 4P' & S1CC'.

Additionally, some groups of images of Fig. 4 and Fig. S1 show the same embryo at different focal planes or show the HCR *in-situ* staining pattern for different mRNAs. Images in the same grouping are of the same embryo: Fig. 4B and Fig. S1C, C', & L; Fig. 4L,L',P,P' and Fig. S1X,X',X",CC,CC' and CC"; Fig. 4C,G and Fig. S1D,D',Q,Q'; Fig. 4O and Fig. S1V,BB,BB'; Fig. S1G and Fig. S1K; Fig. S1J,J' and Fig. S1N,N'; Fig. S1S and Fig. S1Z.

Development • Supplementary information

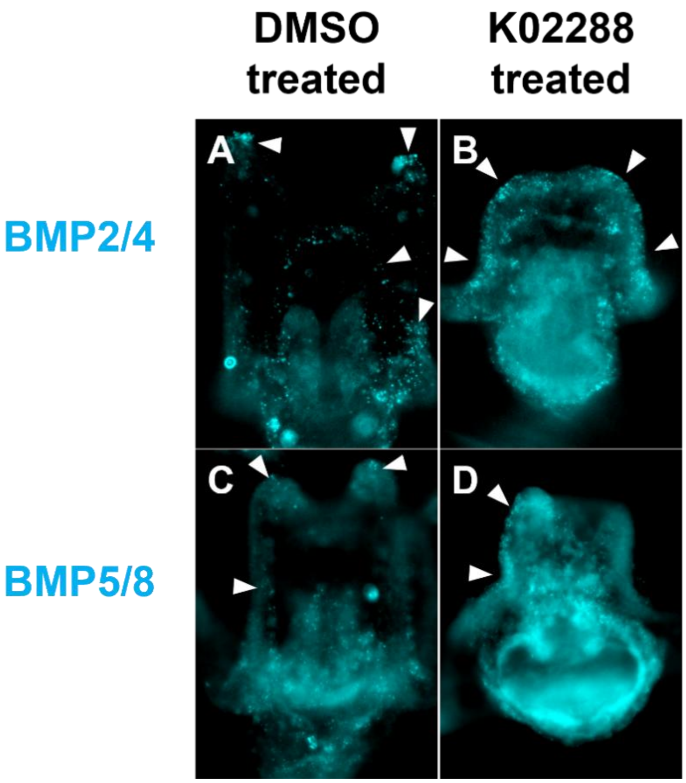

Fig. S2. Spatiotemporal expression pattern of components of the BMP signaling pathway.

L. variegatus embryos treated with DMSO or 1µM K02288 continuously starting after the formation of the tri-radiate spicule rudiments from various stages were fixed at the late pluteus stage (2 days post-fertilization). They were then stained via HCR in-situ hybridization. The two BMP ligands, *bmp2/4* and *bmp5/8*, were normally expressed in the ectoderm of the oral hood in DMSO treated embryos as seen in the untreated embryos in Fig. S1. Additionally, treatment with 1µM K02288 had no effect on expression of either ligand in the ectoderm despite inhibiting expression of both ligands in the DS.

Development • Supplementary information

Table S1. mRNAs with decreased expression following K02288 treatment. Total mRNA from *L. variegatus* embryos treated with DMSO or 1 μM K02288 in ASW continuously starting after the formation of the tri-radiate spicule rudiments was collected at the pluteus stage (2 dpf) and sequenced. This table displays all mRNAs with significantly decreased expression in 1 μM K02288-treated embryos compared to DMSO-treated embryos. See Materials and Methods.

Available for download at
https://journals.biologists.com/dev/article-lookup/doi/10.1242/dev.205344#supplementary-data

Table S2. mRNAs with increased expression following K02288 treatment. Total mRNA from *L. variegatus* embryos treated with DMSO or 1 μM K02288 in ASW continuously starting after the formation of the tri-radiate spicule rudiments was collected at the pluteus stage (2 dpf) and sequenced. This table displays all mRNAs with significantly increased expression in 1 μM K02288-treated embryos compared to DMSO-treated embryos. See Materials and Methods.

Available for download at
https://journals.biologists.com/dev/article-lookup/doi/10.1242/dev.205344#supplementary-data

Table S3. mRNAs with decreased expression following axitinib treatment. Total mRNA from *L. variegatus* embryos treated with DMSO or 75 nM axitinib in ASW continuously starting after the formation of the tri-radiate spicule rudiments was collected at the pluteus stage (2 dpf) and sequenced. This table displays all mRNAs with significantly decreased expression in 75 nM axitinib-treated embryos compared to DMSO-treated embryos. See Materials and Methods.

Available for download at
https://journals.biologists.com/dev/article-lookup/doi/10.1242/dev.205344#supplementary-data

Table S4. mRNAs with increased expression following axitinib treatment. Total mRNA from *L. variegatus* embryos treated with DMSO or 75 nM axitinib in ASW continuously starting after the formation of the tri-radiate spicule rudiments was collected at the pluteus stage (2 dpf) and sequenced. This table displays all mRNAs with significantly increased expression in 75 nM axitinib-treated embryos compared to DMSO-treated embryos. See Materials and Methods.

Available for download at
https://journals.biologists.com/dev/article-lookup/doi/10.1242/dev.205344#supplementary-data

Development • Supplementary information

Table S5. mRNAs similarly impacted by both axitinib and K02288 treatment. This table displays mRNAs with significantly altered expression levels following either drug treatment compared to control embryos. Treatment with one drug resulted in changes to mRNA expression that were not significantly different from treatment with the other drug. See Materials and Methods.

Available for download at
https://journals.biologists.com/dev/article-lookup/doi/10.1242/dev.205344#supplementary-data

Table S6. mRNAs impacted differently by axitinib and K02288 treatment. This table displays mRNAs with significantly altered expression levels following either drug treatment compared to control embryos. Additionally, treatment with one drug resulted in changes to mRNA expression levels that were significantly different from treatment with the other drug. This includes instances where one drug had a more intense effect compared to the other drug, or where two treatments caused opposite changes in mRNA expression levels relative to the base expression level in DMSO treated embryos. See Materials and Methods.

Available for download at
https://journals.biologists.com/dev/article-lookup/doi/10.1242/dev.205344#supplementary-data

Table S7. mRNAs with enriched expression in PMCs.
This table displays mRNAs that were determined to be enriched in PMCs compared to other cell types based on previously published scRNA-seq data (Massri et al., 2021). See Materials and Methods.

Available for download at
https://journals.biologists.com/dev/article-lookup/doi/10.1242/dev.205344#supplementary-data

Table S8. Manually curated mRNAs with known PMC enrichment.

This table contains all mRNAs that were known to be strongly expressed in PMCs or relevant to skeletogenesis based on previous data (see Materials and Methods) which were compared to our RNA-seq data to determine if the expression of these mRNAs was impacted by axitinib or K02288 treatment.

Available for download at
https://journals.biologists.com/dev/article-lookup/doi/10.1242/dev.205344#supplementary-data

Table S9. Comparison of scRNA-seq PMC-enriched mRNA list and drug-affected mRNAs.

This table summarizes the genes identified as PMC enriched in the scRNA-seq that were also identified as affected by either drug in the total RNA-seq experiment performed in this study.

Available for download at
https://journals.biologists.com/dev/article-lookup/doi/10.1242/dev.205344#supplementary-data

Table S10. PMC-enriched mRNAs affected by axitinib and/or K02288.

This table summarizes the findings of comparing known PMC enriched mRNAs to mRNAs identified in the total RNA-seq experiment performed in this study. Enriched mRNAs that were affected by axitinib and/or K02288 are listed with relevant data from the total RNA-seq as well as the impact of drug treatment on mRNA expression. mRNAs are divided into those affected only by K02288, only by axitinib, or affected by both drug treatments relative to DMSO treated embryos.

Available for download at
https://journals.biologists.com/dev/article-lookup/doi/10.1242/dev.205344#supplementary-data

References

DUBOC, V., RÖTTINGER, E., BESNARDEAU, L. & LEPAGE, T. 2004. Nodal and BMP2/4 signaling organizes the oral-aboral axis of the sea urchin embryo. *Dev Cell,* 6**,** 397-410.

MASSRI, A. J., GREENSTREET, L., AFANASSIEV, A., BERRIO, A., WRAY, G. A., SCHIEBINGER, G. & MCCLAY, D. R. 2021. Developmental single-cell transcriptomics in the Lytechinus variegatus sea urchin embryo. Development, 148, dev198614.

PIACENTINO, M. L., RAMACHANDRAN, J. & BRADHAM, C. A. 2015. Late Alk4/5/7 signaling is required for anterior skeletal patterning in sea urchin embryos. *Development,* 142**,** 943-52.

Development • Supplementary information
